# The *Argi* system: one-step purification of proteins tagged with arginine-rich cell-penetrating peptides

**DOI:** 10.1038/s41598-017-02432-6

**Published:** 2017-05-25

**Authors:** Filip Bartnicki, Piotr Bonarek, Ewa Kowalska, Wojciech Strzalka

**Affiliations:** 1 Department of Plant Biotechnology, Faculty of Biochemistry, Biophysics and Biotechnology, Jagiellonian University, Krakow, Poland; 2 Department of Physical Biochemistry, Faculty of Biochemistry, Biophysics and Biotechnology, Jagiellonian University, Krakow, Poland

## Abstract

The discovery of cell penetrating peptides (CPPs) opened new perspectives for the delivery of proteins into human cells. It is considered that in the future CPP-mediated transport of therapeutic proteins may find applications in the treatment of human diseases. Despite this fact a fast and simple method for the purification of CPP-tagged proteins, free of additional tags, was not available to date. To fill this gap we developed the *Argi* system for one-step purification of proteins tagged with arginine rich CPPs.

## Introduction

Cell penetrating peptides (CPPs) are short peptides capable of traversing the plasma membrane. Therefore, they can promote the transport of low and high molecular weight cargos, including peptides and proteins, into the cell^1^. Based on their biochemical properties CPPs are classified into three groups: (i) cationic, (ii) amphipathic and (iii) hydrophobic^2^. Among cationic CPPs a subgroup of arginine rich peptides which can be subdivided into: (i) poly-arginine peptides^3, 4^, (ii) Tat peptides^3^ and (iii) (R-Ahx-R)_4_ peptides^5^, can be distinguished. Poly-arginine CPPs include for example R_6_, or R_8_ peptides composed of six or eight consecutive arginine residues, respectively^4^. One of the Tat peptide variants called Tat_49–57_, which originates from the human immunodeficiency virus (HIV) Tat protein, is composed of nine amino acids including six arginine residues^6^. Finally, the synthetic (R-Ahx-R)_4_ peptide has eight arginine residues which are interspersed with 6-aminohexanoic acid spacers^5^.

Recently, particular attention has been given to studies of potential therapeutic application of CPPs. It is considered that in the future they could play the role of transporters for externally delivered therapeutics, which must cross the plasma membrane to provide the proper functioning of the organism. The advantage of cellular cargo delivery using CPPs is that a broad range of different cell types can uptake these peptides^3^. *In vitro* studies on cationic CPP cytotoxicity showed that they can be tolerated by cells at much higher concentrations than amphipathic CPPs. Although CPP-based therapy has not yet entered human, but only *in vitro* and animal clinical trials, the current results may be cautiously considered as optimistic^7^. There are numerous reports where successful transport of various cargos into different types of cells including plant cells using arginine-rich peptides, was demonstrated^8–14^.

One of the obstacles that limits the potential therapeutic use of arginine-rich CPP-tagged proteins is the lack of appropriate tools dedicated for their purification. For example, up till now CPP-tagged proteins could be purified using multistep conventional chromatography methods, which is laborious, time consuming and may yield a product of unsatisfactory quality and quantity. Alternatively, to provide simple and fast purification of these proteins by affinity chromatography additional tags, e.g. His-tag can be fused with CPP-tagged proteins^8^. However, the additional tag may have a negative impact on the CPP-tagged protein activity. Moreover, in the case of therapeutic applications of the protein the possible induction of human immune response by the additional tag cannot be ignored. For the above reasons the unnecessary extra tag should be removed, which is not always possible, and separated from the protein which significantly increases the cost of large scale CPP-tagged protein production.

The lack of an affinity chromatography system that could be used for efficient purification of arginine rich CPP-tagged proteins is mainly due to the fact that a natural ligand characterized by specific and easily reversible binding of such CPPs was not identified. Therefore, to overcome the current problems with the methodology of arginine-rich CPP-tagged protein purification, minimize the risk of a possible immune response against CPP-tagged therapeutics and to reduce the costs of CPP-tagged protein production in the future, we present here the development of the *Argi* system – a new affinity chromatography tool for one-step purification of arginine-rich CPP-tagged proteins. The presented system is based on the interaction between a DNA aptamer and arginine-rich CPPs.

## Results

### Selection of an arginine-rich CPP-binding aptamer

**Figure 1:**
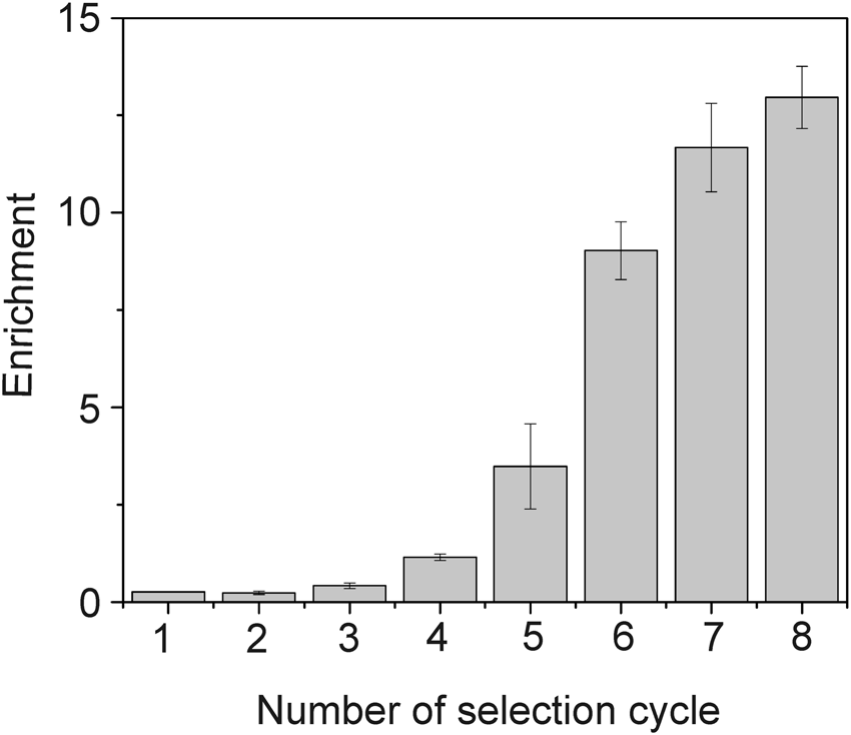
qPCR analysis of aptamer pool binding to R_8_-PCNA. The enrichment of aptamer pools (selection rounds 1–8) was expressed as the ratio: binding of tested aptamer pool/binding of initial ssDNA library to R_8_-PCNA. Results are the mean of three measurements. Error bars represent the standard deviation.

**Figure 2:**
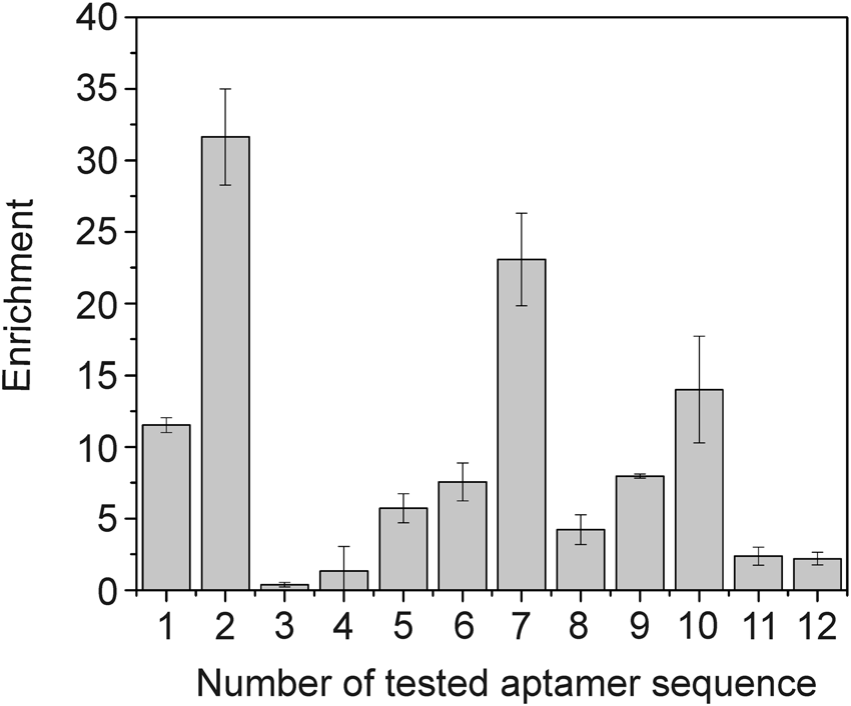
qPCR analysis of selected aptamer binding to R_8_-GST. The enrichment of single aptamers was expressed as the ratio: binding of tested aptamer/binding of reference aptamer to R_8_-GST. Results are the mean of three measurements. Error bars represent the standard deviation.

**Figure 3:**
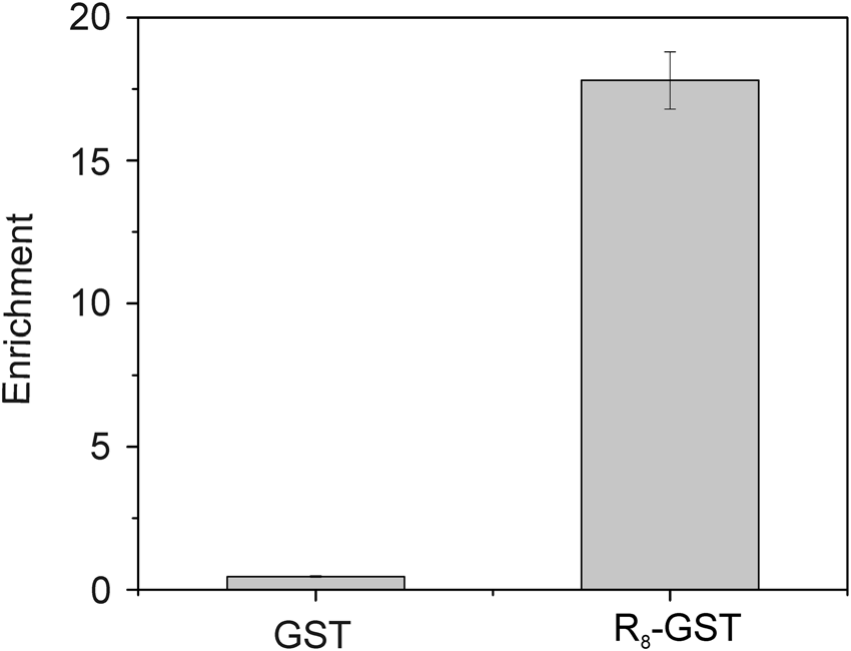
Analysis of 24–10 aptamer specificity using qPCR. The enrichment was expressed as the ratio: binding of tested aptamer/binding of reference aptamer to GST or R_8_-GST. Results are the mean of three measurements. Error bars represent the standard deviation.

To create the *Argi* system we employed systematic evolution of ligands by exponential enrichment (SELEX^15, 16^). A DNA aptamer characterized by specific and reversible binding to poly-arginine and the Tat_49–57_ peptide, fused either to the N- or C- terminus of the tested protein, under mild buffer conditions, was identified. The selection strategy was similar to the one we employed previously^17^. We used a peptide containing eight consecutive arginines (H_6_G_4_R_8_) as a selection target during the first and second round of SELEX. Next, from the third to the eighth selection round R_8_-PCNA was used as a target. The progress of selection was evaluated using qPCR with R_8_-PCNA as a target (Fig. 1). The aptamer pool after the eighth selection round was cloned and sequenced. Among the 50 analyzed clones we found 12 different aptamer sequences. Next, using again qPCR the binding of single aptamers to R_8_-GST was evaluated and compared. The highest value of the enrichment parameter was observed for the aptamer sequence number 2 called 24–10 (Fig. 2) which was selected for further studies. The specificity of this molecule was verified and confirmed (Fig. 3).

**Figure 4:**
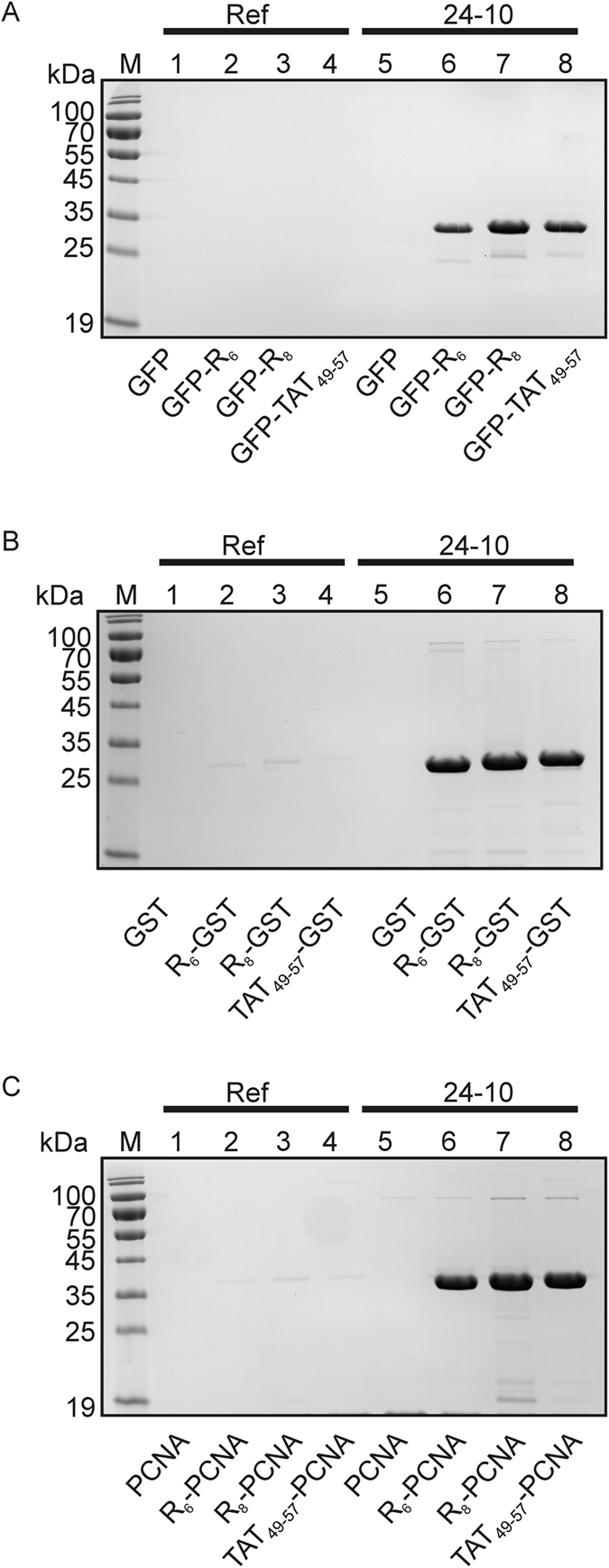
Analysis of the 24–10 aptamer specificity. 5′-biotinylated reference (Ref) (lanes 1–4) or 24–10 (lanes 5–8) aptamer immobilized to streptavidin-agarose resin was incubated with appropriate variants of (**A**) GFP, (**B**) GST and (**C**) PCNA proteins. Protein without tag (lanes 1, 5), protein with R_6_ tag (lanes 2, 6), protein with R_8_ tag (lanes 3, 7), protein with Tat_49–57_ tag (lanes 4, 8). After washing, the protein sample was denatured and half of the protein sample volume of the bound protein was separated on 12% SDS-PAGE gel followed by Coomassie brilliant blue staining. This is one of three independent experiments which is representative.

To test whether the 24–10 aptamer can be used to capture proteins tagged with either the R_6_-tag, R_8_-tag or Tat_49–57_-tag we analyzed and compared the binding of the following proteins, GFP, GFP-R_6_, GFP-R_8_, GFP-Tat_49–57_, GST, R_6_-GST, R_8_-GST, Tat_49–57_-GST, PCNA, R_6_-PCNA, R_8_-PCNA and Tat_49–57_-PCNA, to the 24–10 or reference aptamer immobilized to streptavidin-agarose resin (High Capacity Streptavidin-agarose, HCSA). In line with our expectations, this experiment showed that R_6_-, R_8_- and Tat_49–57_-tagged proteins could be successfully captured only when the 24–10 aptamer was used (Fig. 4, Supplementary Fig. 1).

### Identification of the 24–10 aptamer region necessary for arginine-rich CPP binding

**Figure 5:**
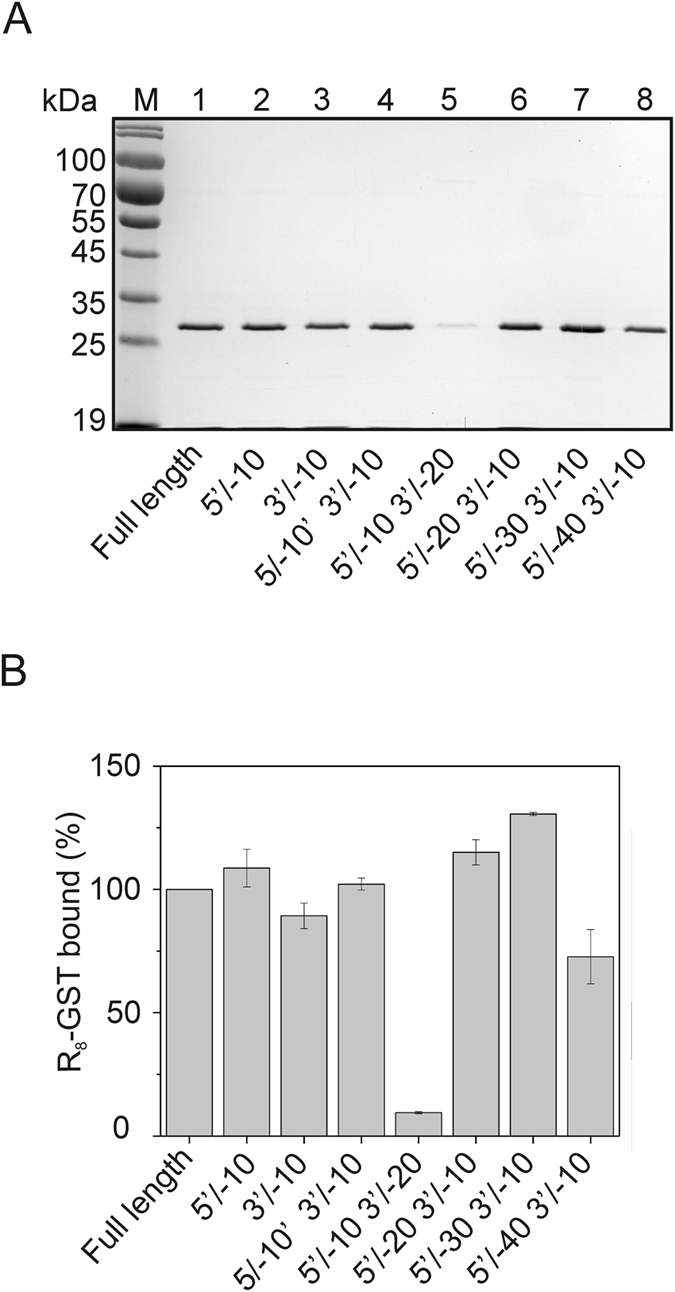
Identification of the 24–10 aptamer region optimal for R_8_ binding. The binding of R_8_-GST to the full length 24–10 aptamer, as well as its variants appropriately shortened from the 5′ or 3′ end, was tested. (**A**) Pull down assay. Lanes: (1) full length 24–10, (2) 24–10 (5′/−10), (3) 24–10 (3′/−10), (4) 24–10 (5′/−10 and 3′/−10), (5) 24–10 (5′/−10 and 3′/−20), (6) 24–10 (5′/−20 and 3′/−10), (7) 24–10 (5′/−30 and 3′/−10) and (8) 24–10 (5′/−40 and 3′/−10) aptamer variants immobilized on streptavidin agarose beads were incubated with the analyzed protein. The bound protein was denatured. Next, one fortieth of the volume of each protein sample was separated on 12% SDS-PAGE followed by Coomassie staining. This is one of three independent experiments which is representative. (**B**) Densitometric analysis of data from three independent pull down assays was done using Multispectral Imaging System IMAGER with Launch VisionWorksLS. Results are the mean of three measurements. The error bars represent standard deviation. The results were normalized relative to signal from the protein sample bound by the full length aptamer (100%).

**Figure 6:**
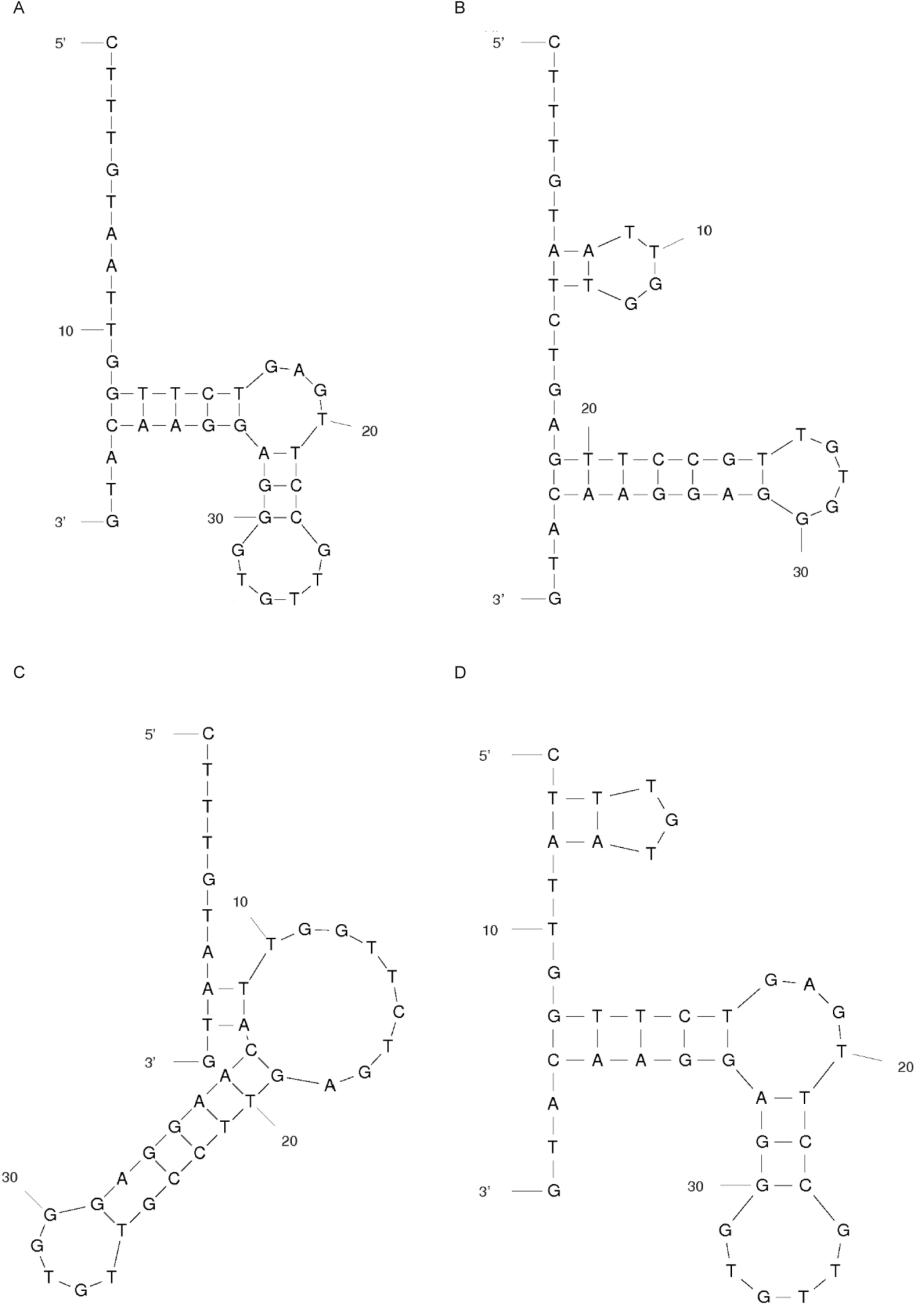
AR aptamer putative secondary structure analysis. The (**A–D**) structures were modeled using mfold web server.

To determine the region of the 24–10 aptamer crucial for binding of the studied arginine-rich CPP tags, R_8_-GST was used as a model. With the help of the pull-down assay the binding of R_8_-GST to variants of the 24–10 aptamer shortened from either the 5′ or 3′ end, as well as the full length aptamer, was evaluated based on densitometric analysis (Fig. 5A,B). This experiment showed that the sequence from nucleotides 31 to 70, referred to as the AR aptamer (5'-CTTTGTAATTGGTTCTGAGTTCCGTTGTGGGAGGAACATG-3'), could bind the R_8_-tag the most efficiently. The modeling of the AR aptamer with mfold^18^ software predicted the formation of four possible structures (Fig. 6). The calculated folding ΔG for structures A, B, C and D was −10.34, −9.86, −9.76 and −9.43 kcal/mol, respectively.

### Analysis of elution conditions for arginine-rich CPP-tagged proteins

**Figure 7:**
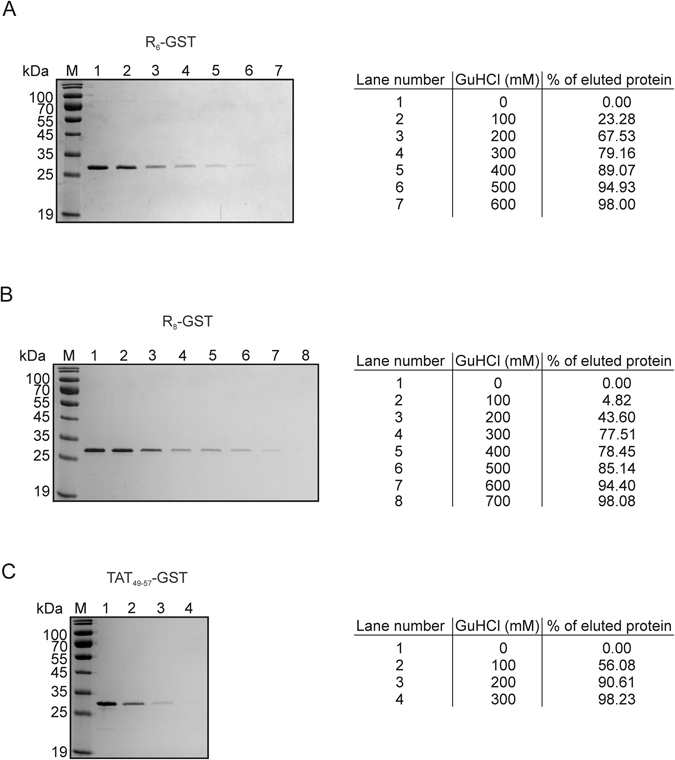
Determination of optimal GuHCl concentration for analyte elution. 5′-biotinylated AR aptamer bound to streptavidin agarose resin was incubated with GST fused with the indicated tag. After washing, the bound protein was eluted using Tris-HCl buffer supplemented with the appropriate GuHCl concentration. Next, the protein samples were denatured and one fortieth of the volume of each sample was separated on 12% SDS-PAGE followed by Coomassie staining and analyzed using Multispectral Imaging System IMAGER with Launch VisionWorksLS. The results were normalized relative to signal from the protein sample eluted with buffer supplemented with 0 mM GuHCl (0% eluted protein). Recombinant GST protein tagged with: (**A**) R_6_, (**B**) R_8_ and (**C**) Tat_49–57_ peptide. Each panel presents the results of one of three independent experiments which is representative.

Among the key features which should characterize affinity chromatography systems, used for recombinant protein purification, the efficient binding of the analyte and its simple elution under mild buffer conditions should be considered. The demonstrated specific interaction between the AR aptamer and tested arginine-rich tags, although crucial, was insufficient to fully assess the possible application of the developed ssDNA for chromatography purposes. This is why in the following experiment the conditions of analyte elution from the AR aptamer were analyzed. Testing buffers supplemented with increasing amounts of guanidine hydrochloride (GuHCl) we showed that to elute more than 90% of R_6_-, R_8_- and Tat_49–57_-GST bound to the AR aptamer, 500, 600 and 200 mM GuHCl, respectively was necessary (Fig. 7A–C). Despite the fact that GuHCl is widely used as a protein denaturant it is well known that at mM concentrations it is usually not harmful but can stabilize proteins^19^.

### Determining the dissociation constant of AR aptamer/arginine-rich CPP complexes

**Figure 8:**
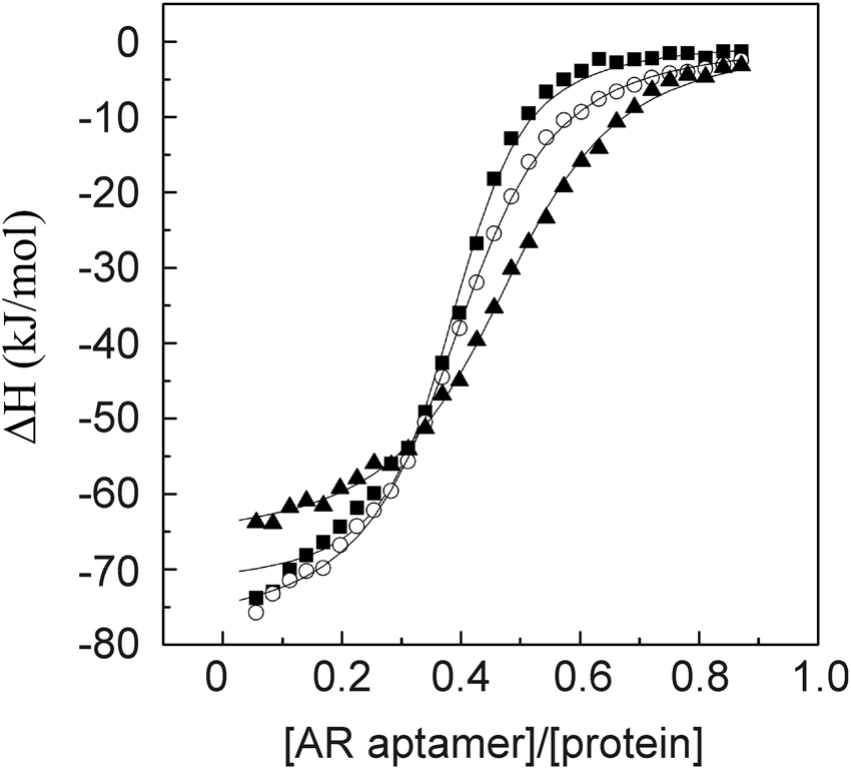
Determination of the AR aptamer/arginine-rich peptide complex dissociation constants. Integrated heats of binding corrected for heats of dilution from titrations of R_8_-GST (squares), R_6_-GST (circles) and Tat_49–57_-GST (triangles) into the solution of AR aptamer at 25 °C. Experiments were performed in 50 mM phosphate buffer (pH 7.6) containing 207 mM NaCl. Solid lines represent the best-fit binding isotherms to the experimental data.

To evaluate the affinity between the developed ssDNA and the studied tags the dissociation constant (Kd) of AR aptamer/arginine-rich tag complexes was determined. The Kd, determined using isothermal titration calorimetry, for the AR aptamer and R_6_-GST, R_8_-GST and Tat_49–57_-GST was 943 ± 37 × 10^−9^ M, 532 ± 55 × 10^−9^ M and 893 ± 44 × 10^−9^ M (Fig. 8), respectively. All reactions were exothermic and enthalpy-entropy driven (Table 1). The analysis of stoichiometry revealed that one molecule of AR-aptamer can bind two molecules of the tested tags.

**Table 1:** Thermodynamic parameters of AR aptamer interaction with R_6_-GST, R_8_-GST and Tat_49–57_-GST determined according to the one set of sites model with assumption that the ligand is in the measurement cell.

	Stoichiometry	ΔG (kJ/mol)	ΔH (kJ/mol)	ΔS (J/mol K)
R_6_-GST	2.61 ± 0.03	−35.8 ± 0.3	−27.7 ± 0.3	27.2 ± 1.9
R_8_-GST	2.51 ± 0.02	−34.4 ± 0.1	−31.0 ± 0.2	11.4 ± 0.8
Tat_49–57_-GST	2.08 ± 0.02	−34.5 ± 0.2	−31.9 ± 0.2	8.9 ± 1.1

### Evaluation of AR aptamer application for purification of CPP-tagged proteins

**Figure 9:**
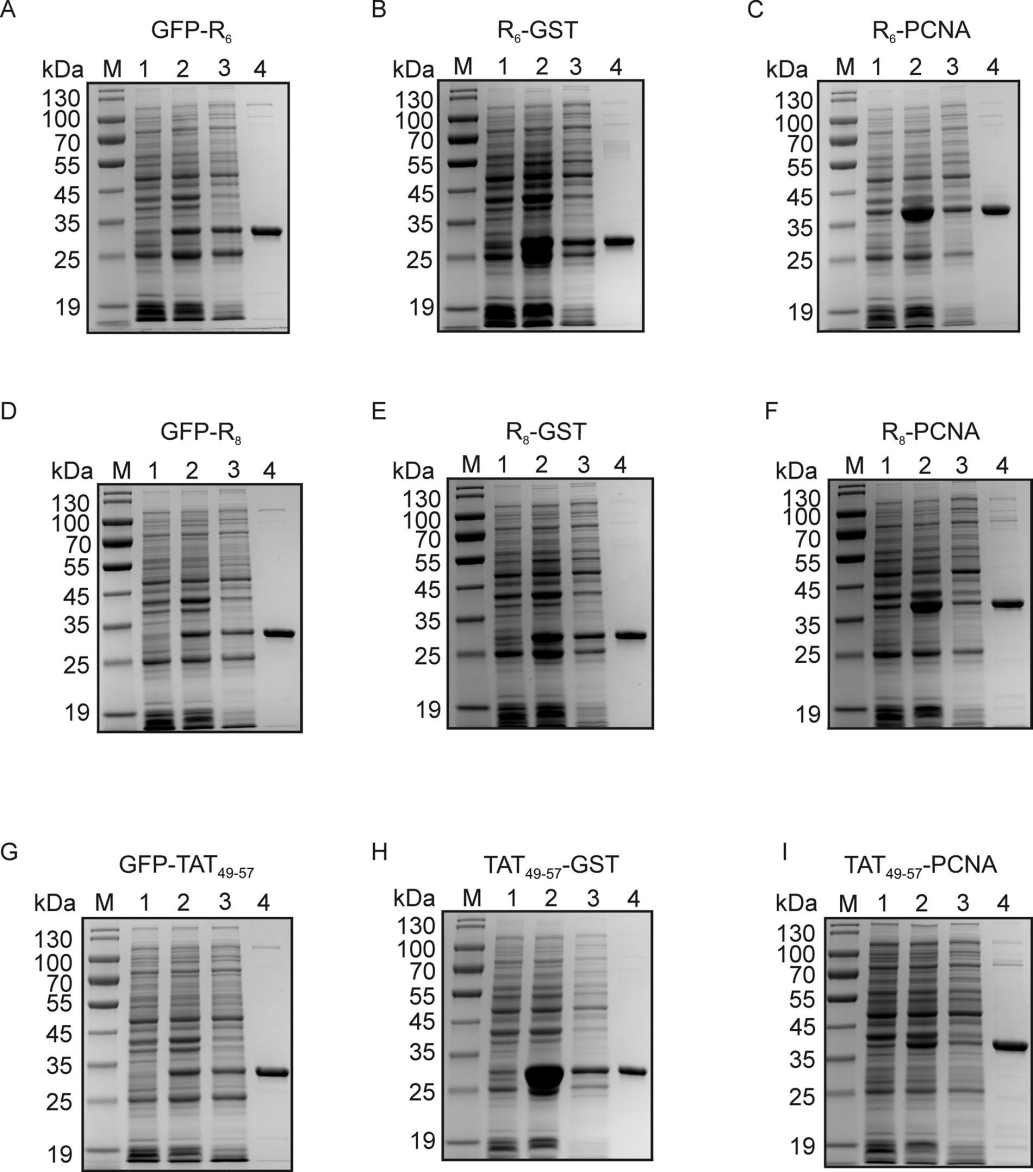
Analysis of arginine-rich peptide-tagged protein purification from *E. coli* total protein extract using AR aptamer-based chromatography. The recombinant proteins: GFP-R_6_ (**A**), R_6_-GST (**B**), R_6_-PCNA (**C**), GFP-R_8_ (**D**), R_8_-GST (**E**), R_8_-PCNA (**F**), GFP-Tat_49–57_ (**G**), Tat_49–57_-GST (**H**) and Tat_49–57_-PCNA (**I**) were overexpressed and purified from *E. coli* total protein extract using AR aptamer. Lanes: M) molecular weight marker; 1) non-induced BL21-CodonPlus(DE3)-RIL[pET29a arginine-rich peptide-tagged protein] cells; 2) induced BL21-CodonPlus(DE3)-RIL [pET29a arginine-rich peptide-tagged protein] cells; 3) cell lysate; 4) 4 μg of protein sample eluted from AR aptamer-based resin using ARGI buffer supplemented with GuHCl. The samples were denatured and separated on 12% SDS-PAGE followed by Coomassie brilliant blue staining. This is one of three independent experiments which is representative.

**Figure 10:**
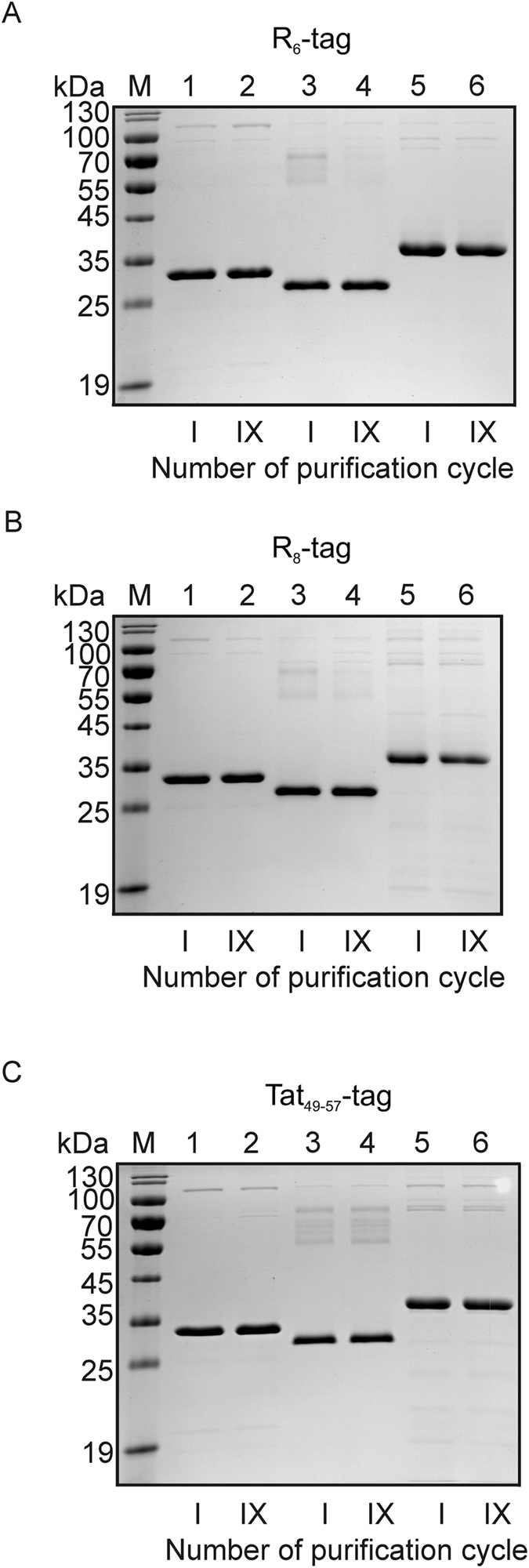
The analysis of AR aptamer-based chromatography reproducibility. 5′-biotinylated AR aptamer bound to streptavidin-agarose resin was incubated with *E. coli* total protein extracts containing recombinant proteins. After washing, the bound protein was eluted using buffer supplemented with an appropriate GuHCl concentration. (**A**) R_6_, (**B**) R_8_ and (**C**) Tat_49–57_ tagged recombinant protein. Lanes: 1 and 2) GFP-tag, 3 and 4) tag-GST, 5 and 6) tag-PCNA. The purification of the tested protein was repeated nine times using the same resin. Next, 4 μg of protein sample eluted after the first and ninth purification cycle was denatured and separated on 12% SDS-PAGE followed by Coomassie staining. This is one of three independent experiments which is representative.

Finally, when the biochemical studies of the *Argi* system were completed, to test the developed tool in real conditions the purification of nine different protein variants (GFP-R_6_, GFP-R_8_, GFP-Tat_49–57_, R_6_-GST, R_8_-GST, Tat_49–57_-GST, R_6_-PCNA, R_8_-PCNA and Tat_49–57_-PCNA) from *E. coli* total protein extract was performed and analyzed. These experiments clearly confirmed the applicability of the developed AR aptamer for the purification of recombinant proteins tagged with arginine-rich peptides (Fig. 9). Moreover, we found that using 175 μg of AR aptamer we could purify from 145 to 594 μg of protein (Supplementary Table 1). Additionally, analyzing the reproducibility of the *Argi* system we found that during nine purification cycles the quality of isolated proteins was not significantly affected (Fig. 10).

## Discussion

In this study we presented the development of the *Argi* system which is a powerful tool for one-step purification of recombinant proteins tagged with arginine-rich cell-penetrating peptides overexpressed in *E. coli* cells. Besides the application of poly-arginine peptides for cargo transport, in 1984 the utilization of this tag for the purification of recombinant proteins was proposed^20^, but has not found a wide application in laboratories. The purification of arginine-rich peptide-tagged proteins was based on the use of non-specific ion exchange chromatography. However, the pH of the buffers used during purification was far from the pH range which is usually optimal for protein stability and activity. The applied buffer conditions could possibly be harmful for the biological activity of many proteins. On the other hand other studies demonstrated that the poly-arginine tag could improve protein solubility^21^. Despite previous reports, an affinity chromatography system for the purification of poly-arginine-tagged proteins was never developed. Therefore, the presented new chromatography tool offers a much easier method of arginine rich CPP-tagged protein purification, which was not available up to date.

## Materials and Methods

### Experimental

#### Chemicals and plasmids

The chemicals used in this work were obtained from Sigma-Aldrich (USA) and Merck (Germany) unless indicated otherwise. Synthetic aptamers, the ssDNA library and reference aptamer (5′-CATGCTTCCCCAGGGAGATGACTGACTGACTGACTGACTGACTGACT GACTGACTGACTGGAGGAACATGCGTCGCAAAC-3′) were obtained from IBA GmbH (Germany). The template plasmids pDNR-LIB (coding for human PCNA), pGEX4T-1 (coding for GST) and pK7WGF2 (coding for GFP) were purchased from ImaGenes GmbH, GE HealthCare and VIB Department of Plant Systems Biology, University of Ghent, respectively.

#### Protein immobilization

The H_6_G_4_R_8_ peptide (LifeTein, USA) was dissolved in DMSO to a concentration of 1 mg/mL. Next, 10 μg of the peptide was mixed with 0.5 μL 50% cobalt-coated agarose beads (TALON, Clontech) and the mixture was incubated in AS1 buffer (136 mM NaCl, 12 mM KCl, 10 mM Na_2_HPO_4_, 1.7 mM KH_2_PO_4_, 4.9 mM MgCl_2_, 0.01% (v/v) Tween 20; pH 7.5) for 1 h at RT with continuous mixing. To remove the unbound peptide, after immobilization the beads were washed 3x with AS1 buffer. Immobilization of R_8_-GST, GST or R_8_-PCNA to CNBr-activated Sepharose 4B (Pharmacia) was performed according to the protocol supplied by the manufacturer.

#### Cloning and plasmid construction

Nucleotide sequences coding for GFP-R_6_/R_8_/Tat_49–57_, R_8_/Tat_49–57_-GST and R_8_/Tat_49–57_-PCNA were amplified using sets of specific primers (Supplementary Table 2) and cloned into the pET29a vector as described previously^17^ with minor modifications. The following PCR conditions were used: preliminary denaturation at 94 **°**C for 2 min, 30 cycles of amplification (94 °C for 40 s, 58 °C for 30 s, 72 °C for 3 min), final incubation at 72 °C for 7 min. The PCR products were purified, digested with BamHI and NdeI (FastDigest, Thermo Scientific) restriction enzymes and cloned into the pET29a expression vector using T4 DNA ligase (Thermo Scientific), followed by sequencing. The construction of *E. coli* expression vectors carrying PCNA- and GFP-coding sequences was previously described^17^. Open reading frames coding for R_6_-GST and R_6_-PCNA were synthetized by Genscript.

#### Protein overexpression

The GFP, GFP**-**R_6_/R_8_/ Tat_49–57_, GST, R_6_/R_8_/ Tat_49–57_-GST, PCNA, and R_6_/R_8_/ Tat_49–57_-PCNA proteins were overexpressed as described previously for GFP, GST and PCNA^17^.

#### Protein purification

All the purification steps were performed at 4 °C similarly as described previously^17^. The *E. coli* cells containing all variants of GFP, GST and PCNA were resuspended in GFP buffer (50 mM Tris-HCl, 3 M NaCl, pH 8.0), PBS(140 mM NaCl, 2.7 mM KCl, 10 mM Na_2_HPO_4_,1.8 mM KH_2_PO_4_, pH 7.3) buffer and PCNA buffer (50 mM Tris-HCl, pH 7.6), respectively. Phenylmethylsulfonyl fluoride (PMSF) was then added to 1 mM final concentration. Next, the samples were sonicated (10 min, 5 s pulses, 10 s break) and centrifuged (30,000x g for 30 min) to obtain *E. coli* total protein extracts. Next, the proteins were purified as described below. At the final step of protein purification the fractions containing the protein of interest were pooled, dialyzed against a S buffer (50 mM Tris-HCl, 150 mM NaCl, 15% (v/v) glycerol, pH 7.5), frozen in liquid nitrogen and stored at −80 °C until use.

#### GFP and GFP-R_6_/R_8_/ Tat_49–57_ purification

The protein extract was loaded onto a 5 mL HiTrap Butyl HP column (GE Healthcare) equilibrated with GFP buffer. The unbound proteins were washed out with GFP buffer. Next, the bound proteins were eluted using 200 mL of linear gradient from 3–0 M NaCl. To obtain the satisfactory protein purity, the fractions containing the protein of interest were dialysed against PCNA buffer and loaded onto a 5 mL High Q (Bio-Rad) column. After washing with PCNA buffer the bound proteins were eluted using 200 mL of linear gradient from 0–0.8 M NaCl in PCNA buffer.

#### GST and R_6_/R_8_/ Tat_49–57_-GST purification

The protein extract was loaded onto a 2 mL Glutathione Sepharose 4B column (GE Healthcare) equilibrated with PBS buffer. The unbound proteins were washed out with PBS buffer. Next, the bound protein was eluted with GST elution buffer (50 mM Tris-HCl, 50 mM reduced glutathione, pH 8.0).

#### PCNA and R_6_/R_8_/ Tat_49–57_-PCNA purification

The proteins were purified according to the protocol described previously for PCNA and His_3_-PCNA^17^.

### SELEX procedure

The SELEX procedure was performed as described previously^17^ with some modifications. AS1 buffer was used for aptamer selection. The H_6_G_4_R_8_ peptide bound to TALON cobalt-coated agarose beads (Clontech), which were shown previously to provide good selection power^22^, was used for selection rounds I and II. For selection cycles III to VIII R_8_-PCNA, immobilized to to CNBr-activated Sepharose 4B (Pharmacia), was used as a target molecule.

### Quantitative real-time PCR

qPCR analysis of aptamer binding, enrichment and specificity was performed as described previously^17^. R_8_-PCNA was used as a target when the enrichment parameter was analyzed for the aptamer pool after each SELEX cycle. R_8_-GST was used to test the enrichment of particular aptamer sequences. R_8_-GST and GST were used to evaluate the specificity of the 24–10 aptamer.

### Pull-down-based assays

Assays were performed at 4 °C using a universal spin column (MoBiTec) and High Capacity Streptavidin Agarose (HCSA) resin (ThermoScientific). After each washing or elution step, the resin was incubated for 5 min before centrifugation (800xg for 30 s).

#### Pull-down assay

10 μL of 50% (w/v) HCSA resin was washed with water and AS1 buffer. Next it was incubated with 500 pmoles of the 5′-biotinylated Ref or 24–10 aptamer in 300 μL of AS1 buffer for 1 h at RT with gentle agitation. Next, the beads were washed (3 × 500 μL with AS1 buffer if not stated otherwise) and incubated with GFP, GST, PCNA fused with R_6_/R_8_/Tat_49–57_ or with proteins without any tag (final concentration 4 μM) in 500 μl of AS1 buffer at 4 °C for 1 h. The resin was then washed 5 × 500 μL with AS1 buffer to wash out unbound protein. The aptamer-bound protein was denatured with 1 × GLB buffer (50 mM Tris-HCl, 2% (w/v) SDS, 2% (w/v) bromophenol blue (w/v), 10% (v/v) glycerol, 200 mM β-mercaptoethanol, pH 6.8) at 95 °C for 5 min. Next, half of the protein sample volume was separated in 12% SDS-PAGE^23^, followed by Coomassie brilliant blue staining.

#### Determination of the 24–10 aptamer fragment essential for arginine-rich peptide tag binding

The experiment was performed as described in section (Pull-down assay) with minor modifications. AS1 buffer was replaced with ARGI buffer (50 mM Tris-HCl, 300 mM NaCl, 5 mM EDTA, 0.01% (v/v) Tween 20, pH 7.5). 20 μL of 50% (w/v) HCSA resin coupled with 1 nmole of the 5′-biotinylated full length 24–10 aptamer (5′-CATGCTTCCCCAGGGAGATGGACGGCACGTCTTTGTAATTGGTTCTG AGTTCCGTTGTGGGAGGAACATGCGTCGCAAAC-3′) or its variants shortened either from the 5′ or 3′ end were incubated with R_8_-GST (final concentration 4 μM). After washing out the unbound protein 5 × 500 μL with ARGI buffer the aptamer-bound protein was denatured with 1 × GLB at 95 °C for 5 min. Next, one fortieth of the volume of each protein sample was separated on 12% SDS-PAGE followed by Coomassie brilliant blue staining.

#### Determination of protein elution conditions

The experiment was performed as described in section (Determination of the 24–10 aptamer fragment essential for arginine-rich peptide tag binding) with minor modifications. 20 μL of 50% (v/v) HCSA resin coupled with 1 nmole of the 5′-biotinylated AR aptamer (5′-CTTTGTAATTGGTTCTGAGTTCCGTTGTGGGAGGAACATG-3′) was incubated with the R_6_/R_8_ or Tat_49–57_-GST protein (final concentration 4 μM) in 500 μl of ARGI buffer. After washing out the unbound protein the bound protein was eluted with ARGI buffer (5 × 500 μL) supplemented with different concentrations of guanidine hydrochloride (GuHCl), in the following ranges: 0–600 mM for R_6_-GST, 0–700 mM for R_8_-GST and 0–300 mM for Tat_49–57_-GST. The aptamer-bound protein was denatured with 1 × GLB at 95 °C for 5 min. Next, one fortieth of the volume of each protein sample was separated on 12% SDS-PAGE followed by Coomassie brilliant blue staining.

#### Purification of R_6_/R_8_/ Tat_49–57_-tagged proteins using the AR aptamer

The experiment was performed as described in section (Pull-down assay) with minor modifications. AS1 buffer was replaced with ARGI buffer. 14 nmoles of the 5′-biotinylated AR aptamer were immobilized using 150 μL of 50% (v/v) HCSA resin. After washing, the resin was incubated for 1 h with gentle agitation with 1 ml of *E. coli* total protein extract (final concentration 10 mg/mL) prepared similarly as described in section (Protein purification), in ARGI buffer. Unbound proteins were washed out with ARGI buffer, and the bound protein was eluted by rinsing the resin 5 × with ARGI buffer supplemented with appropriate concentrations of GuHCl. The eluted protein was concentrated using Amicon Ultra-0.5(Merck Millipore Ltd.) and dialyzed against S buffer without glycerol. The Bradford protein assay (Bio-Rad) was used to measure protein concentration in the samples which were next denaturated at 95 °C for 5 min and separated on 12% SDS-PAGE, followed by Coomassie brilliant blue staining.

#### Reproducibility of AR aptamer-based chromatography

The experiment was performed as described in section (Purification of R6/R8/Tat49-57-tagged proteins using the AR aptamer) with minor modifications. After each purification procedure the HCSA-AR aptamer resin was regenerated with R buffer (50 mM Tris-HCl, 1 M NaCl, 1 M GuHCl, 0.01% (v/v) Tween 20, pH 7.5). When not used the HCSA-AR aptamer resin was stored in S buffer at −80 °C.

### Dissociation constant determination

The isothermal titration calorimetry experiments were carried out in duplicates at 25 °C using a VP-ITC instrument (MicroCal, Northampton, MA, USA). Typically, 30 injections of 4 µL aliquots of 200 µM AR aptamer were added into a 1.4355 ml calorimeter cell containing 20 µM tagged protein in 50 mM phosphate buffer (pH 7.6) containing 207 mM NaCl. The injection speed was 0.5 µL/s with 4 min intervals between injections. All solutions were degassed under vacuum prior to use in ITC experiments. In order to ensure proper mixing after each injection, a constant stirring speed of 300 rpm was maintained during the experiment. The heat of the AR aptamer dilution was used to correct the total heat of binding prior to data analysis. The nonlinear analysis was performed according to the one set of sites model using Origin7 software.

### Computational Analysis

The models of AR aptamer secondary structure and Δ*G* were calculated using the mfold web server^18^. The calculation was performed for the following conditions: temperature 25 °C and 300 mM NaCl.

## Electronic supplementary material

Supplementary Information
