# Supplementary material for: The Argi system: one-step purification of proteins tagged with arginine-rich cell-penetrating peptides
Source: Sci Rep. 2017 May 25;7:2619. doi: 10.1038/s41598-017-02432-6 (PMC5453957; doi:10.1038/s41598-017-02432-6)
Supplement: Supplementary file 1 — Supplementary Information [file 41598_2017_2432_MOESM1_ESM.pdf]

The *Argi* system: one-step purification of proteins tagged with arginine-rich cell-penetrating peptides

Filip Bartnicki[1], Piotr Bonarek[2], Ewa Kowalska[1], Wojciech Strzalka[1]*

Supplementary Figure 1.

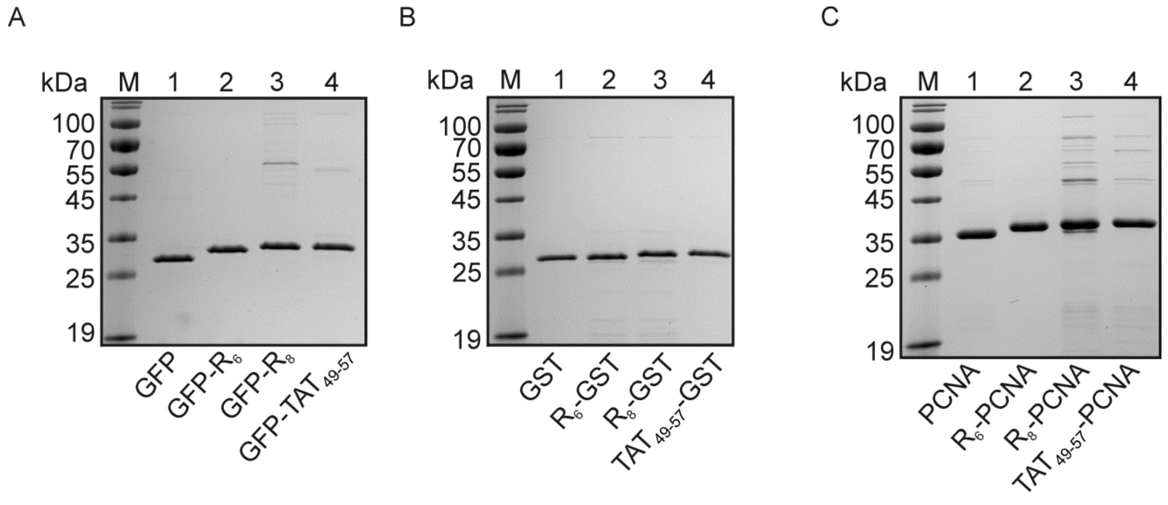

Supplementary Figure. 1. Input proteins used in the experiments

Appropriate variants of (A) GFP, (B) GST and (C) PCNA were used in this study. Lanes: 1) protein without tag, 2) protein with R_6 tag, 3) protein with R_8 tag, 4) protein with Tat_{49-57} tag. Two µg of analyzed protein was denatured and separated on 12% SDS-PAGE gel followed by Coomassie staining.

**Protein name**	**Quantity of purified protein (µg)**
GFP-R_6	196
R_6-GST	440
R_6-PCNA	260
GFP-R_8	386
R_8-GST	594
R_8-PCNA	151
GFP-Tat_{49-57}	292
Tat_{49-57}-GST	565
Tat_{49-57}-PCNA	145

Supplementary Table 1. Exemplary amounts of recombinant proteins purified from *E. coli* total extracts containing 10 mg of total protein. The resin with 175 µg of AR aptamer was used for purification.

ORF	Forward primer
GFP	5'-ttccatatggtgagcaagggcgaggagctgttc-3'
GFP-Arg_6	5'-ttccatatggtgagcaagggcgaggagctgttc-3'
GFP-Arg_8	5'-ttccatatggtgagcaagggcgaggagctgttc-3'
GFP-Tat_{49-57}	5'-ttccatatggtgagcaagggcgaggagctgttc-3'
PCNA	5'-ggaattccatatgttcgaggcgcgcctggtccagggctc-3'
Arg_8-PCNA	5'-ggaattccatatgcgccgtcgccgtcgccgtcgccgtggtagcgatagcatgttcgaggcgcgcctggtccagggctc-3'
Tat_{49-57}-PCNA	5'-ttccatatgcgcaaaaaacgccgtcagcgtcgccgtggtggcggtggcggtggccatccatggatgttcgaggcgcgcctggtccag-3'
Arg_8-GST	5'-ggaattccatatgcgccgtcgccgtcgccgtcgccgtggtagcgatagcatgtcccctatactagg-3'
Tat_{49-57}-GST	5'-ttccatatgcgcaaaaaacgccgtcagcgtcgccgtagccaggacccgatgtcccctatactagg-3'
**ORF**	**Reverse primer**
GFP	5'-tctggatccttacttgtacagctcgtccatgccgagagtg-3'
GFP-Arg_6	5'-tctggatccttagcgacggcgacggcgacgaccgccaccgctatcgctcttgtacagctcgtccatgccgagagtg-3'
GFP-Arg_8	5'-tctggatccttaacggcgacggcgacggcgacggcgaccgccagaatgtggccacttgtacagctcgtccatgccgagagtg-3'
GFP-Tat_{49-57}	5'-tctggatccttagcgtttttttgcgacgctgacggcgacgaccgccagaatgtggccacttgtacagctcgtccatgccgagagtg-3'
PCNA	5'-gcggatccctaagatccttcttcatcctcgatcttg-3'
Arg_8-PCNA	5'-gcggatccctaagatccttcttcatcctcgatcttg-3'
Tat_{49-57}-PCNA	5'-gcggatccctaagatccttcttcatcctcgatcttg-3'
Arg_8-GST	5'-gcggatcctcaacgcggaaccagatccg-3'
Tat_{49-57}-GST	5'-gcggatcctcaacgcggaaccagatccg-3'

Supplementary Table 2. List of used primers.
